# Interferon-Inducible E3 Ligase RNF213 Facilitates Host-Protective Linear and K63-Linked Ubiquitylation of Toxoplasma gondii Parasitophorous Vacuoles

**DOI:** 10.1128/mbio.01888-22

**Published:** 2022-09-26

**Authors:** Dulcemaria Hernandez, Stephen Walsh, Luz Saavedra Sanchez, Mary S. Dickinson, Jörn Coers

**Affiliations:** a Department of Molecular Genetics and Microbiology, Duke University Medical Center, Durham, North Carolina, USA; b Department of Immunology, Duke University Medical Center, Durham, North Carolina, USA; Stanford University

**Keywords:** LUBAC, RNF213, TAX1BP1, *Toxoplasma*, cell-autonomous immunity, interferons, linear ubiquitin, mysterin, parasitology, parasitophorous vacuole, ubiquitination, xenophagy

## Abstract

The obligate intracellular protozoan pathogen Toxoplasma gondii infects a wide range of vertebrate hosts and frequently causes zoonotic infections in humans. Whereas infected immunocompetent individuals typically remain asymptomatic, toxoplasmosis in immunocompromised individuals can manifest as a severe, potentially lethal disease, and congenital *Toxoplasma* infections are associated with adverse pregnancy outcomes. The protective immune response of healthy individuals involves the production of lymphocyte-derived cytokines such as interferon gamma (IFN-γ), which elicits cell-autonomous immunity in host cells. IFN-γ-inducible antiparasitic defense programs comprise nutritional immunity, the production of noxious gases, and the ubiquitylation of the *Toxoplasma*-containing parasitophorous vacuole (PV). PV ubiquitylation prompts the recruitment of host defense proteins to the PV and the consequential execution of antimicrobial effector programs, which reduce parasitic burden. However, the ubiquitin E3 ligase orchestrating these events has remained unknown. Here, we demonstrate that the IFN-γ-inducible E3 ligase RNF213 translocates to *Toxoplasma* PVs and facilitates PV ubiquitylation in human cells. *Toxoplasma* PVs become decorated with linear and K63-linked ubiquitin and recruit ubiquitin adaptor proteins in a process that is RNF213 dependent but independent of the linear ubiquitin chain assembly complex (LUBAC). IFN-γ priming fails to restrict *Toxoplasma* growth in cells lacking RNF213 expression, thus identifying RNF213 as a potent executioner of ubiquitylation-driven antiparasitic host defense.

## INTRODUCTION

*Toxoplasma* is an endoparasite of the Apicomplexa family that includes *Plasmodium*, the causative agent of malaria (1). *Toxoplasma* infects a broad range of mammalian and avian hosts, but the only known definitive host for *Toxoplasma* is cats. In the feline gut epithelium, *Toxoplasma* undergoes a sexual replication cycle and forms oocysts, which are shed in cat feces. Following ingestion of oocysts by a host organism, sporozoites contained within oocysts differentiate into asexually replicating tachyzoites. Fast-growing tachyzoites convert into slow-growing and cyst-forming bradyzoites in specific tissue environments, and the ingestion of food contaminated with cysts is an additional mechanism by which definitive and intermediate hosts become infected with *Toxoplasma* (1, 2). Zoonotic *Toxoplasma* infections are frequent in humans but generally asymptomatic in immunocompetent individuals. However, severe opportunistic infections can occur in immunodeficient adults, including AIDS patients, and congenital infections can lead to miscarriages, preterm births, and stillbirths (3–5).

Mice, a major prey of many cat species, are common intermediate hosts of *Toxoplasma* (1). Indeed, compelling evidence indicates that molecular arms races between components of the innate immune system of mice and specific *Toxoplasma* virulence factors have shaped the evolution of mouse and *Toxoplasma* genomes alike (6–9). Therefore, mice serve as a biologically relevant model to study mechanisms of host defense to *Toxoplasma* infections, and much of what we know about the immune response to *Toxoplasma* infections has been gleaned from experimental work conducted in laboratory mice. Based on this large body of work, it is now well established that interleukin 12 (IL-12) cytokine production by immune sentinel cells and the resulting interferon gamma (IFN-γ) secretion by IL-12-responsive innate and adaptive lymphocytes are central to the ability of mice to fight off *Toxoplasma* infections (10–13). To exert host defense, secreted IFN-γ binds to its cognate IFN-γ receptor, which is expressed on virtually all cells of the mammalian body, thereby inducing robust expression of hundreds of IFN-γ-inducible genes (ISGs). Proteins encoded by ISGs facilitate cell-autonomous immunity through the execution of diverse antimicrobial activities (12, 13).

In mice, ISGs encoding families of IFN-inducible dynamin-related GTPases play dominant roles in cell culture models of cell-autonomous immunity and overall host protection against *Toxoplasma in vivo* (14). Specifically, mice lacking expression of discrete members of the immunity-related GTPase (IRG) or the guanylate-binding protein (GBP) families succumb to infections with *Toxoplasma* strains that are nonlethal in isogenic wild-type (WT) animals (15–21). IRGs and GBPs execute host defense by trafficking to *Toxoplasma* parasitophorous vacuoles (PVs), where these GTPases distort PV membranes and ultimately rupture PVs to expel the parasite into the host cell cytosol (15, 22–24). Once exposed to the cytosolic milieu of the host cell, *Toxoplasma* is again attacked by specific IRG proteins, which bind directly to the parasite and appear to strip *Toxoplasma* of its plasma membrane, thus facilitating rapid parasite killing (22).

IRGs and GBPs work cooperatively in the cell-autonomous immune response to *Toxoplasma* in mouse cells and have been shown to promote each other’s recruitment to PVs (12–14). The mechanism by which IRGs and GBPs are delivered to *Toxoplasma* PVs is only poorly understood but likely involves the concerted action of multiple events, including the attachment of ubiquitin-like Atg8 proteins to PV membranes through a noncanonical autophagy-related process, recognition of missing-self patterns, the sensing of specific lipid species enriched in PV membranes, and the ubiquitylation of PV membranes (21, 25–32). Members of the murine M clade of IRG (IRGM) proteins facilitate the delivery of effector IRGs to PV membranes and are also essential for PV ubiquitylation in mouse cells (28, 30). Moreover, the specific effector Irgb6 is essential for PV ubiquitylation (21). These observations indicate that effector IRGs, such as Irgb6, shuttle ubiquitin E3 ligases to PV membranes. However, effector IRGs are absent from human cells (14, 33), which nonetheless ubiquitylate PVs in response to IFN-γ stimulation (34–36), similar to murine cells. Therefore, the mechanisms of IFN-γ-inducible PV ubiquitylation are likely distinct in murine and human hosts.

Members of the TRAF family of ubiquitin E3 ligases localize to *Toxoplasma* PVs in mouse and human cells and enhance PV ubiquitylation and cell-autonomous immunity to *Toxoplasma* in IFN-γ-primed cells (28, 37). Similarly, the ubiquitin E3 ligase Trim21 traffics to *Toxoplasma* PVs in murine cells and conveys immune protection to *Toxoplasma* infections *in vivo.* However, PV ubiquitylation is only moderately reduced in Trim21-deficient mouse cells, and the defect is limited to polyubiquitin chains linked at lysine residue 63 (K63) (38). TRIM21 also facilitates PV ubiquitylation in human fibroblasts infected with an avirulent type III strain of *Toxoplasma* but not in cells infected with a virulent type II strain that expresses high levels of the secreted virulence factor ROP18, a kinase targeting TRIM21 for lysosomal degradation (39–41).

In addition to K63-linked ubiquitin, IFN-γ priming also leads to the decoration of *Toxoplasma* PVs with linear ubiquitin (M1 linked) (34), which is defined as ubiquitin molecules linked via the C-terminal carboxyl group of a donor ubiquitin and the N-terminal methionine (M1) of an acceptor ubiquitin (42). The only known ubiquitin E3 ligase-catalyzing linear ubiquitin chain formation is the linear ubiquitin chain assembly complex (LUBAC) composed of its essential components, SHARPIN, HOIL-1, and HOIP (43–45). Previous work demonstrated that Hoil-1-deficient mice display increased susceptibility to *Toxoplasma* infections *in vivo*, but whether LUBAC is required for linear ubiquitylation of *Toxoplasma* PVs was not reported (46). Here, we show that LUBAC is unexpectedly dispensable for linear ubiquitylation of *Toxoplasma* PVs in human cells. Instead, we observe that the IFN-γ-inducible ubiquitin E3 ligase RNF213 translocates to *Toxoplasma* PVs and facilitates K63- and M1-linked ubiquitylation of the *Toxoplasma* PV. We further show that human cells lacking RNF213 expression are defective for IFN-γ-mediated cell-autonomous immunity to *Toxoplasma*. Collectively, our study characterizes RNF213 as a central executioner of antiparasitic PV ubiquitylation in human cells.

## RESULTS

### LUBAC is dispensable for *Toxoplasma* PV ubiquitylation and cell-autonomous host defense.

**FIG 1:**
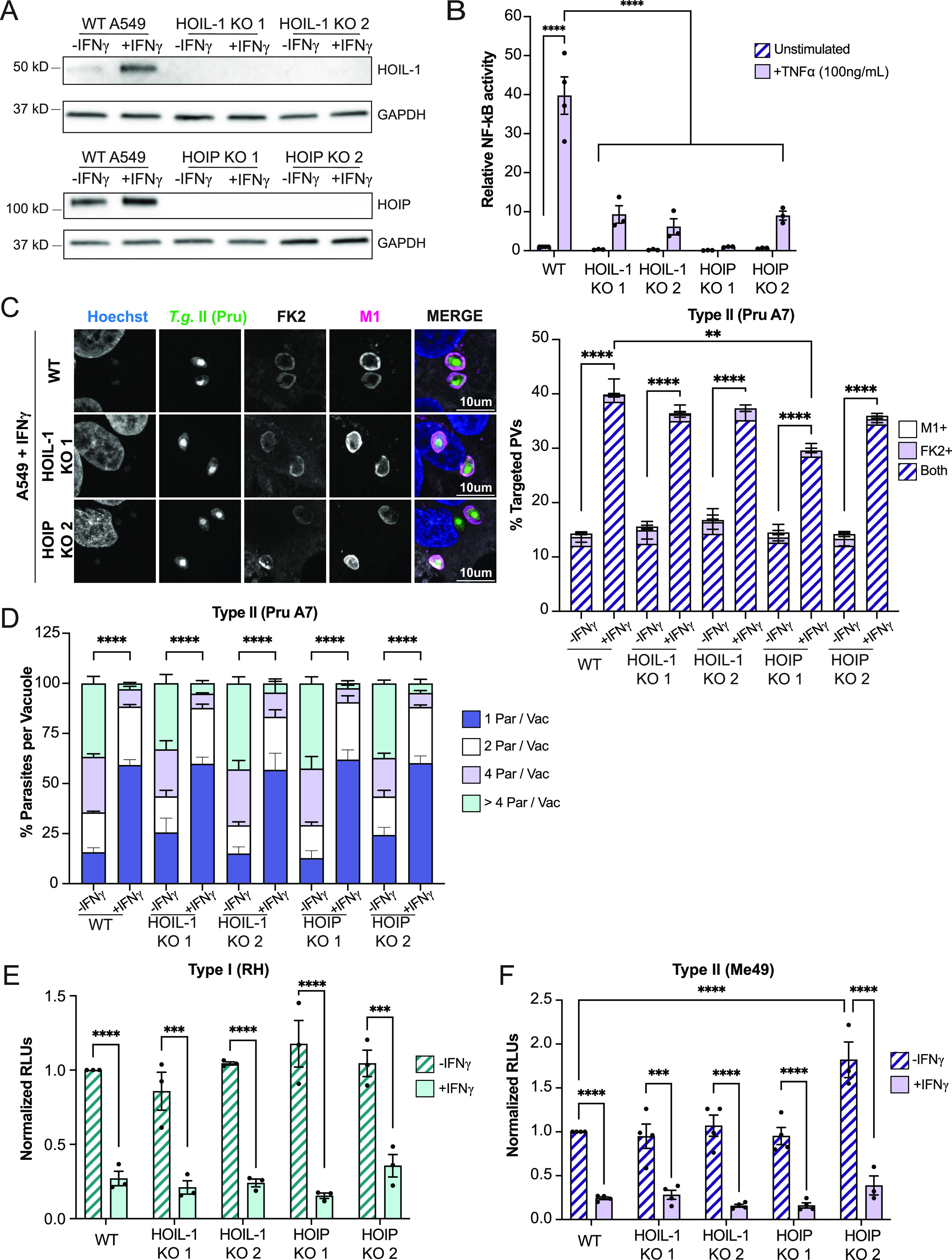
The LUBAC complex is dispensable for IFN-γ-mediated *Toxoplasma* PV ubiquitylation and clearance. (A) Western blotting showing absence of HOIP and HOIL-1 expression in CRISPR-generated deletion (KO) A549 cells. (B) WT, HOIP KO, and HOIL-1 KO A549 cells were cotransfected with NF-κB-Luc and hRluc/TK reporter constructs. At 24 h posttransfection, cells were left unprimed or primed with TNF-α (100 ng/mL) for 24 h, and cell extracts were analyzed for luciferase activity. (C) Unprimed or IFN-γ-primed (100 U/mL) WT, HOIP KO, and HOIL-1 KO A549 cells were infected with *Toxoplasma* type II (Pru) at an MOI of 3 for 6 h. Representative confocal images and quantification of ubiquitin (FK2) and M1 recruitment to PVs are shown. Statistical comparisons are shown for groups “both.” (D) Unprimed or IFN-γ-primed (100 U/mL) WT, HOIP KO, and HOIL-1 KO A549 cells were infected at an MOI of 2 with *Toxoplasma* type II (Pru), and parasites per vacuole were quantified at 24 hpi. (E and F) Unprimed and IFN-γ-primed (100 U/mL) WT A549, HOIP KO, and HOIL-1 KO cells were infected with luciferase-expressing strains RH and Me49 at an MOI of 1 or MOI of 2, respectively, and cell lysates were analyzed for luciferase activity at 24 hpi. Growth of each strain was normalized to growth in WT unprimed cells. All data depict the mean ± SEM from 3 to 4 independent experiments. Two-way ANOVA followed by Tukey’s multiple-comparison test was used to determine significance. *, *P* < 0.05; **, *P* < 0.01; ***, *P* < 0.001; ****, *P* < 0.0001; ns, not significant.

Linear (M1) ubiquitylation of bacterial pathogens residing inside the host cell cytosol has emerged as a cell-autonomous hosted defense mechanism executed by the E3 ubiquitin ligase LUBAC (47). Because *Toxoplasma* PVs are M1 ubiquitylated in IFN-γ-primed human cells (34), we hypothesized that LUBAC was facilitating *Toxoplasma* PV ubiquitylation and associated host defenses. We opted to test this hypothesis in the human lung epithelial cell line, A549, which was previously validated as a robust model for IFN-γ-dependent *Toxoplasma* PV ubiquitylation and restriction of parasitic growth (28, 35, 48–50). We therefore generated deletion (knockout [KO]) mutants in two genes encoding the essential LUBAC components HOIP and HOIL-1 in A549 cells and validated the KOs by Western blotting (Fig. 1A). Additionally, we demonstrated that HOIP and HOIL-1 KO cells were defective for tumor necrosis factor alpha (TNF-α)-induced activation of the proinflammatory transcription factor NF-κB (Fig. 1B), a signaling cascade known to be LUBAC dependent (51, 52).

Next, we used these validated KO cell lines to determine whether LUBAC plays a role in PV ubiquitylation. In these studies, we infected cells with the type II *Toxoplasma* strain Pru, which was previously shown to be susceptible to PV ubiquitylation (28, 30, 34, 36, 37). Confirming these reported observations, we found that IFN-γ priming led to increased Pru PV ubiquitylation in wild-type (WT) A549 cells. Unexpectedly, the percentages of PVs staining positive for anti-pan-ubiquitin or M1-linked ubiquitin remained largely unchanged in cells lacking functional LUBAC, except for a moderate reduction in ubiquitylated PVs in one out of two HOIP KO clonal cell lines, likely due to some clonal variation (Fig. 1C). In agreement with the immunofluorescence data, HOIP and HOIL-1 KO cells maintained their ability to restrict growth of *Toxoplasma* type II strains Pru and Me49, as well as type I strain RH, in response to IFN-γ priming (Fig. 1D to F). In summary, these data demonstrated that LUBAC is dispensable for IFN-γ-mediated M1 ubiquitylation of PVs and IFN-γ-inducible cell-autonomous immunity.

### IFN-γ-inducible ubiquitin E3 ligase RNF213 colocalizes with *Toxoplasma* PVs.

**FIG 2:**
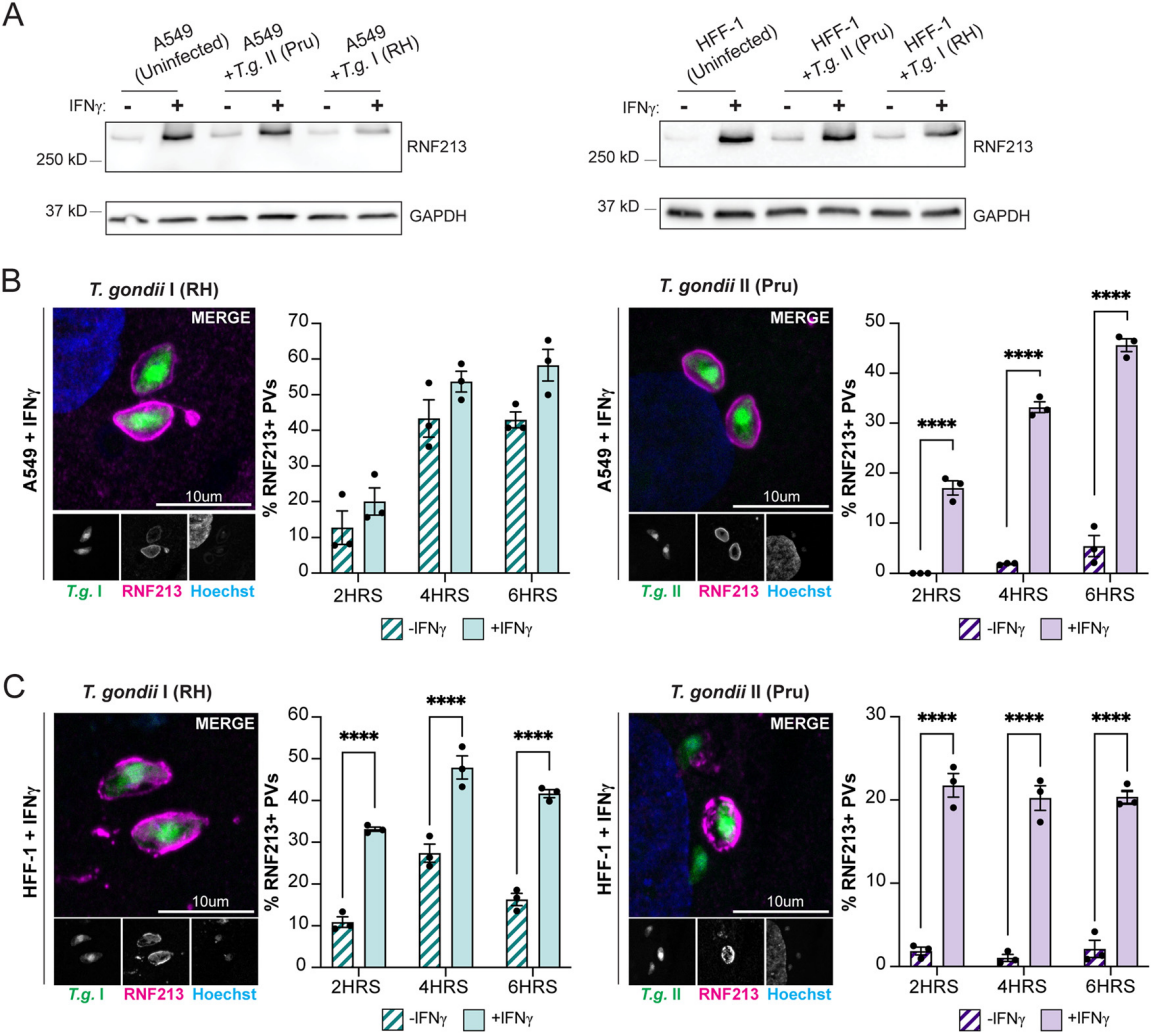
The IFN-γ-inducible E3 ligase RNF213 colocalizes with *Toxoplasma* PVs in A549 and HFF-1 cells. (A) Western blotting showing expression of RNF213 during infection in unprimed or IFN-γ-primed (100 U/mL) WT A549 and HFF-1 cells. Cells were infected with *Toxoplasma* strains RH or Pru at an MOI of 3, and protein lysates were collected at 6 hpi. (B and C) Representative confocal images and quantification of RNF213 recruitment to *Toxoplasma* RH and Pru PVs in WT A549 and HFF-1 cells. Unprimed or IFN-γ-primed (100 U/mL) WT A549 and HFF-1 cells were infected with RH or Pru at an MOI of 3. RNF213 recruitment was quantified at 2 hpi, 4 hpi, and 6 hpi. All data depict the mean ± SEM from 3 independent experiments. Two-way ANOVA followed by Tukey’s multiple-comparison test was used to determine significance. *, *P* < 0.05; **, *P* < 0.01; ***, *P* < 0.001; ****, *P* < 0.0001; ns, not significant.

Recent work characterized the ubiquitin E3 ligase RNF213 as a novel antiviral and antibacterial protein (53–56). In line with its role as a host defense protein, we observed that RNF213 protein expression, already detectable in naive cells, was further induced by IFN-γ priming in A549 and a human foreskin fibroblast cell line (HFF-1) (Fig. 2A). Because RNF213 was recently shown to bind to Gram-negative and Gram-positive bacteria invading the host cell cytosol (53, 54), we asked whether RNF213 could similarly associate with *Toxoplasma* PVs. Confirming this hypothesis, we found that up to nearly 60% of *Toxoplasma* PVs immunostained positive for RNF213 within the first 6 h postinfection (hpi) in both IFN-γ-primed A549 and HFF-1 cells infected with either type I RH or type II Pru *Toxoplasma* (Fig. 2B and C). Overall, RNF213 targeting efficiency to PVs was moderately higher in A549 than in HFF-1 cells, and notably, PVs formed by RH *Toxoplasma* colocalized more frequently with RNF213 than PVs containing Pru, especially in unprimed cells (Fig. 2B and C). The recruitment of RNF213 to *Toxoplasma* PVs occurred independent of the host defense proteins ISG15 and GBP1 (Fig. S1A and B), two proteins previously shown to restrict *Toxoplasma* replication in A549 cells (48, 50). Therefore, we can exclude a model in which RNF213 translocation to PVs is controlled by GBP1 or the known RNF213 interaction partner ISG15 (54).

10.1128/mbio.01888-22.1 FIG S1 RNF213 is recruited to *Toxoplasma* PVs independent of ISG15 and GBP1. (A and B) Western blotting for ISG15 and GBP1 protein expression in unprimed and IFN-γ-primed (100 U/mL) WT, ISG15 KO, and GBP1 KO A549 cells. (C and D) Unprimed and IFN-γ-primed (100 U/mL) WT, ISG15 KO, and GBP1 KO A549 cells were infected with *Toxoplasma* type II Pru at an MOI of 3, and RNF213 recruitment to *Toxoplasma* PVs was quantified at 6 hpi. All data depict the mean ± SEM from 3 independent experiments. Two-way ANOVA followed by Tukey’s multiple-comparison test was used to determine significance. *, *P* < 0.05; **, *P* < 0.01; ***, *P* < 0.001; ****, *P* < 0.0001; ns, not significant. Download FIG S1, PDF file, 0.2 MB. Copyright © 2022 Hernandez et al. 2022 Hernandez et al. https://creativecommons.org/licenses/by/4.0/ This content is distributed under the terms of the Creative Commons Attribution 4.0 International license.

### RNF213 facilitates M1- and K63-linked *Toxoplasma* PV ubiquitylation and the recruitment of ubiquitin adaptors.

**FIG 3:**
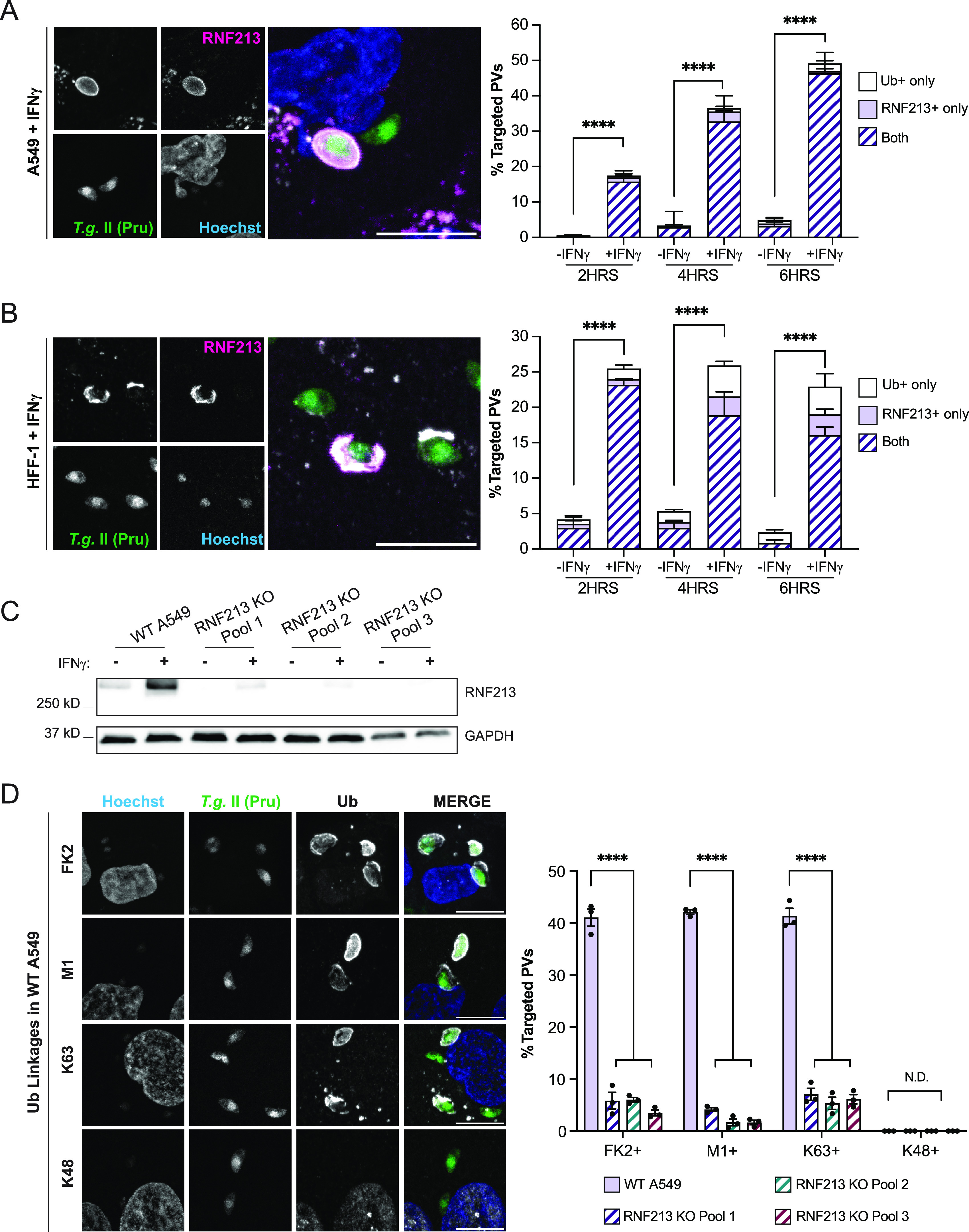
RNF213 colocalizes with ubiquitin at the *Toxoplasma* PV and facilitates M1- and K63-linked ubiquitylation. (A and B) Representative confocal images of RNF213 and ubiquitin (FK2) localization to *Toxoplasma* type II Pru PVs in IFN-γ-primed WT A549 (A) and HFF-1 (B) cells at 6 hpi. Colocalization was quantified at 2 hpi, 4 hpi, and 6 hpi, and combined data from three independent experiments are shown. Statistical comparisons are shown for groups “both.” (C) Western blotting for RNF213 expression in unprimed and IFN-γ-primed (100 U/mL) WT deletion (KO) A549 pools. (D) Representative confocal images of ubiquitin (FK2) and ubiquitin linkages M1, K63, and K48 found on the PV in WT A549 cells at 6 hpi. Quantification of ubiquitin linkages detectable on *Toxoplasma* Pru PVs in IFN-γ-primed (100 U/mL) WT and RN213 KO A549 cells infected at an MOI of 3 for 6 h. All data depict the mean ± SEM from 3 independent experiments. Two-way ANOVA followed by Tukey’s multiple-comparison test was used to determine significance. *, *P* < 0.05; **, *P* < 0.01; ***, *P* < 0.001; ****, *P* < 0.0001; ns, not significant.

To investigate a potential functional role for RNF213 in PV ubiquitylation, we infected unprimed and IFN-γ-primed A549 and HFF-1 cells with *Toxoplasma* and immunostained cells for RNF213 and ubiquitin. We observed that nearly all RNF213-positive PVs were also ubiquitylated, both in the case of infections with *Toxoplasma* type II strain Pru (Fig. 3A and B) and with type I strain RH (see Fig. S2A and B in the supplemental material). It is notable that frequent RNF213 translocation to PVs occurred and coincided with PV ubiquitylation also in unprimed cells infected with *Toxoplasma* type I strain RH (Fig. S2A and B). Although the kinetics and magnitude of ubiquitin-RNF213 colocalization varied with the specific *Toxoplasma* strain and host cell used, collectively, these data supported the hypothesis that RNF213 could facilitate PV ubiquitylation. To test this hypothesis directly, we generated three independent pools of RNF213 KO A549 cells. We obtained high-efficiency genome editing with all three independent small guide RNAs (>95% based on surveyor assays), which led to the depletion of RNF213 expression in the three corresponding RNF213 KO pools, as assessed by Western blotting (Fig. 3C). To avoid the compounding effects of interclonal phenotypic heterogeneity (57), we forewent the subcloning of individual KO lines and instead used RNF213 KO pools for our subsequent studies.

10.1128/mbio.01888-22.2 FIG S2 RNF213 colocalizes with ubiquitin on *Toxoplasma* RH PVs. Unprimed and IFN-γ-primed (100 U/mL) WT A549 (A) and HFF-1 (B) cells were infected with *Toxoplasma* type I (RH) at an MOI of 3, and RNF213 recruitment to PVs and colocalization with ubiquitin (FK2) were quantified at 2 hpi, 4 hpi, and 6 hpi. Statistical comparisons are shown for groups “both.” Representative confocal images at 6 hpi are shown. All data depict the mean ± SEM from 3 to 4 independent experiments. Two-way ANOVA followed by Tukey’s multiple-comparison test was used to determine significance. *, *P* < 0.05; **, *P* < 0.01; ***, *P* < 0.001; ****, *P* < 0.0001; ns, not significant. Download FIG S2, PDF file, 0.8 MB. Copyright © 2022 Hernandez et al. 2022 Hernandez et al. https://creativecommons.org/licenses/by/4.0/ This content is distributed under the terms of the Creative Commons Attribution 4.0 International license.

We observed the near-complete loss of *Toxoplasma* PV ubiquitylation in IFN-γ-primed RNF213 KO cells (Fig. 3D). Because the few ubiquitylated PVs detectable in RNF213 KO pools also stained positive for RNF213 (Fig. S3), we can conclude that this residual PV ubiquitylation is likely attributable to the small percentage of WT A549 cells contained within the KO pool populations. Confirming previous observations (34), we were able to detect M1- and K63-linked, but not K48-linked, ubiquitin on *Toxoplasma* PVs. Like total ubiquitin, the attachment of M1- and K63-linked ubiquitin to PVs was RNF213 dependent (Fig. 3D), demonstrating an essential role for RNF213 in LUBAC-independent linear ubiquitylation of PVs.

10.1128/mbio.01888-22.3 FIG S3 Rare ubiquitin-positive PVs costain for RNF213 due to “contaminating” WT cells present in RN213 KO pools. Unstimulated or IFN-γ-primed (100 U/mL) WT and RNF213 KO pool A549 cells were infected with *Toxoplasma* type II (Pru) at an MOI of 3. RNF213 recruitment to the PV and ubiquitin (FK2) colocalization was quantified at 6 hpi. Statistical comparisons are shown for “both” groups. All data depict the mean ± SEM from 3 independent experiments. Two-way ANOVA followed by Tukey’s multiple-comparison test was used to determine significance. *, *P* < 0.05; **, *P* < 0.01; ***, *P* < 0.001; ****, *P* < 0.0001; ns, not significant. Download FIG S3, PDF file, 0.1 MB. Copyright © 2022 Hernandez et al. 2022 Hernandez et al. https://creativecommons.org/licenses/by/4.0/ This content is distributed under the terms of the Creative Commons Attribution 4.0 International license.

**FIG 4:**
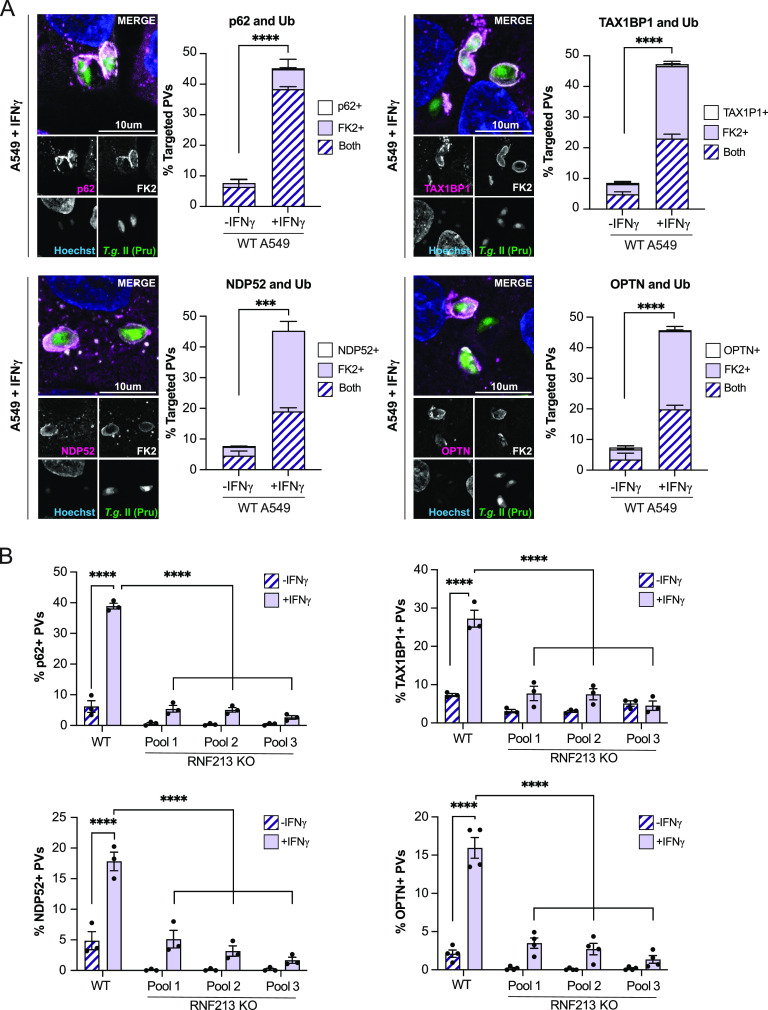
RNF213 facilitates recruitment of ubiquitin adaptors to the *Toxoplasma* PV. (A) Representative confocal images and quantification of ubiquitin adaptors (p62, TAX1BP1, NDP52, and OPTN) and ubiquitin (FK2) colocalization to *Toxoplasma* Pru PVs in WT A549 cells. Unprimed and IFN-γ-primed (100 U/mL) cells were infected with *Toxoplasma* Pru at an MOI of 3 for 6 h. Statistical comparisons is shown for groups “both.” (B) Unprimed and IFN-γ-primed (100 U/mL) WT and RN213 KO A549 cells were infected with *Toxoplasma* Pru at an MOI of 3, and the localization of p62, TAX1BP1, NDP52, and OPTN to *Toxoplasma* PVs was quantified at 6 hpi. All data depict the mean ± SEM from 3 to 4 independent experiments. Two-way ANOVA followed by Tukey’s multiple-comparison test was used to determine significance. *, *P* < 0.05; **, *P* < 0.01; ***, *P* < 0.001; ****, *P* < 0.0001; ns, not significant.

The specific fate of ubiquitylated *Toxoplasma* PVs varies with human cell types but is generally dependent on the action of ubiquitin-binding proteins (34, 35, 58), which localize to ubiquitylated PVs and orchestrate downstream host defense pathways. Confirming and expanding upon previous results (34, 35, 50, 58), we detected the recruitment of the ubiquitin adaptor proteins p62, TAX1BP1, NDP52, and OPTN exclusively to ubiquitylated *Toxoplasma* PVs. This colocalization of ubiquitin-binding proteins with *Toxoplasma* type II Pru PVs increased by up to 5-fold with IFN-γ priming (Fig. 4A), indicating that an IFN-γ-inducible cofactor facilitates the delivery of these proteins to PVs. Confirming this hypothesis, we noticed the near-complete loss of PV-resident ubiquitin adaptor proteins in RNF213 KO cell pools (Fig. 4B), demonstrating the essential function of IFN-γ-inducible RNF213 in delivering antimicrobial ubiquitin-binding proteins to *Toxoplasma* PVs.

### RNF213 executes antiparasitic activity in IFN-γ-primed A549 cells.

**FIG 5:**
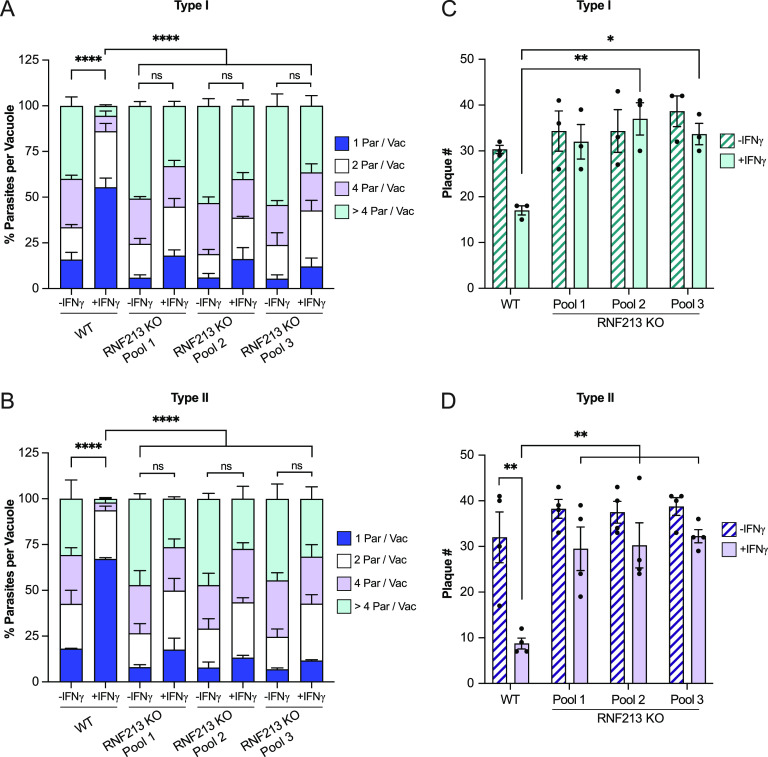
RNF213 mediates restriction of *Toxoplasma* in IFN-γ-primed A549. (A and B) Unprimed and IFN-γ-primed (100 U/mL) WT and RNF213 KO A549 cells were infected with *Toxoplasma* type I RH and type II Pru at an MOI of 1 or MOI of 2, respectively, and parasites per vacuole were quantified at 24 hpi. Statistical comparisons are shown for groups “1 parasite per vacuole.” (C and D) *Toxoplasma* plaque number in unprimed or IFN-γ-primed (100 U/mL) WT and RNF213 KO A549 cell pools. Cell monolayers in 96-well plates were infected with 50 *Toxoplasma* RH or Pru tachyzoites per well, and plaque formation was quantified at 72 hpi. All data depict the mean ± SEM from 3 to 4 independent experiments. Two-way ANOVA followed by Tukey’s multiple-comparison test was used to determine significance. *, *P* < 0.05; **, *P* < 0.01; ***, *P* < 0.001; ****, *P* < 0.0001; ns, not significant.

Whereas IFN-γ-primed murine cells lyse *Toxoplasma* PVs and kill parasites ejected into the host cell cytosol, IFN-γ-primed human nonhematopoietic cells impede *Toxoplasma* growth within the parasite’s vacuolar niche (13, 59). In agreement with previous reports, the majority of *Toxoplasma* PVs inside IFN-γ-primed WT A549 cells contained only a single parasite per vacuole, and only 5.5% of RH PVs and 2% of Pru PVs contained more than 4 parasites at 24 hpi, a dramatic reduction in parasitic burden compared to unprimed WT cells (Fig. 5A and B). In contrast to WT cells, IFN-γ priming failed to significantly reduce *Toxoplasma* burden in RNF213 KO cells, as enumerated by measuring parasites per vacuole (Fig. 5A and B). The loss of cell-autonomous immunity in RNF213-deficient cells was confirmed by plaque assays, through which we assessed lytic growth of RH and Pru strain tachyzoites in cell monolayers over the course of 72 h (Fig. 5C and D), and by measuring relative light units emitted by luminescent type I RH and type II Me49 strains (Fig. S4A and B). Collectively, these data characterize the ubiquitin E3 ligase RNF213 as a potent executioner of human antiparasitic host defense.

10.1128/mbio.01888-22.4 FIG S4 RNF213 mediates restriction of *Toxoplasma* type I RH and type II Me49. Unprimed and IFN-γ-primed (100 U/mL) WT and RNF213 KO A549 cells were infected with luciferase-expressing strains RH (A) and Me49 (B) at an MOI of 1 or MOI of 2, respectively, and cell lysates were analyzed for luciferase activity at 24 hpi. Growth of each strain in IFN-γ-primed cells was normalized to growth in unprimed cells. All data depict the mean ± SEM from 3 to 4 independent experiments. One-way ANOVA followed by Tukey’s multiple-comparison test was used to determine significance. *, *P* < 0.05; **, *P* < 0.01; ***, *P* < 0.001; ****, *P* < 0.0001; ns, not significant. Download FIG S4, PDF file, 0.2 MB. Copyright © 2022 Hernandez et al. 2022 Hernandez et al. https://creativecommons.org/licenses/by/4.0/ This content is distributed under the terms of the Creative Commons Attribution 4.0 International license.

## DISCUSSION

A central driver of cell-autonomous immunity to *Toxoplasma* is the lymphocyte-derived cytokine IFN-γ. IFN-γ priming results in the ubiquitylation of the *Toxoplasma* PV in human epithelial cells and other cell types (34–37), yet no ubiquitin E3 ligase that is essential for this process was previously reported. Our study identifies the IFN-γ-inducible ubiquitin E3 ligase RNF213 as an essential orchestrator of *Toxoplasma* PV ubiquitylation and associated host defense in human A549 epithelial cells. Cells lacking RNF213 expression neither ubiquitylate *Toxoplasma* PVs nor launch a measurable antiparasitic response upon IFN-γ priming. Translocation of RNF213 to *Toxoplasma* PVs also occurs in fibroblasts, another cell type we examined, thus suggesting that RNF213 plays a central role in antiparasitic cell-autonomous immunity, likely across many human cell types.

Confirming previous results (34), we observed that IFN-γ priming prompted the buildup of K63- and M1-linked ubiquitin chains on *Toxoplasma* PVs. The only previously identified E3 ligase for M1-linked ubiquitin chain formation is LUBAC (43–45). Because recent work reported that RNF213-mediated ubiquitylation of bacterial outer membrane lipopolysaccharide (LPS) resulted in LUBAC recruitment and linear ubiquitylation of Gram-negative bacteria in the host cell cytosol (53), we anticipated a similar functional relationship between RNF213 and LUBAC in the context of *Toxoplasma* PV ubiquitylation. Unexpectedly, linear ubiquitylation of PVs also occurred in cells lacking essential components of LUBAC. Notably, our study did not determine whether the ubiquitin ligase activity of RNF213 itself catalyzes the formation of linear ubiquitin chains, and we cannot rule out that RNF213 operates in concert with an unidentified linear ubiquitin E3 ligase (Fig. S5). Therefore, testing the ability of RNF213 to form M1-linked ubiquitin chains *in vitro* and identifying the ubiquitylated substrates on PV membranes are important avenues of future research to shed some light on these unexpected findings.

10.1128/mbio.01888-22.5 FIG S5 Schematic for RNF213-mediated restriction of *Toxoplasma* growth. RNF213 localizes to *Toxoplasma* PVs and facilitates K63-linked and linear PV ubiquitylation. This process is independent of LUBAC but may still require additional ubiquitin E3 ligases. Ubiquitin adaptor proteins p62, TAX1BP1, NDP52, and OPTN associate with ubiquitylated PVs. RNF213-mediated PV ubiquitylation is unlikely to be sufficient for restriction of *Toxoplasma* growth but, rather, appears to require additional ISGs. Created with BioRender.com. Download FIG S5, PDF file, 0.6 MB. Copyright © 2022 Hernandez et al. 2022 Hernandez et al. https://creativecommons.org/licenses/by/4.0/ This content is distributed under the terms of the Creative Commons Attribution 4.0 International license.

As already stated, we observed that RNF213 facilitates the formation of both M1- and K63-linked ubiquitin chains on PV membranes. The functional importance of each of these two types of ubiquitin chains present on *Toxoplasma* PVs is currently unclear and will require additional experimental work to disentangle. Importantly, our data indicate that RNF213-dependent PV ubiquitylation is not sufficient to restrict parasitic growth. This conclusion is based on our observation that PVs formed by type I *Toxoplasma* strain RH become heavily targeted by RNF213 and ubiquitylated not only in IFN-γ-primed but also in unprimed cells. However, IFN-γ priming remains essential for RNF213-dependent cell-autonomous immunity against RH *Toxoplasma*, and therefore, additional IFN-γ-inducible factors must operate synergistically with RNF213 to reduce parasitic burden (see Fig. S5 in the supplemental material). While we favor a model in which RNF213 executes its antiparasitic activity through PV ubiquitylation, RNF213 bound to PVs may execute other modalities of host defense that are independent of ubiquitylation.

There are many additional questions emanating from our findings. Whereas the targeting of RNF213 to cytosolic Gram-negative bacteria is readily explained by the ability of RNF213 to bind to LPS (53), *Toxoplasma* or its surrounding PV lack LPS, and therefore, RNF213 must be recruited to PVs through an LPS-independent mechanism. Further considering the fact that RNF213 can also associate with the Gram-positive and, thus, LPS-free bacterium Listeria monocytogenes in the host cell cytosol and has also been shown to execute antiviral activities (54, 55), it appears that RNF213 protein is equipped with the ability to detect a broad spectrum of phylogenetically diverse intracellular pathogens. The underlying molecular mechanisms of this broadly active immune recognition by RNF213 are unknown. Differences between *Toxoplasma* strains may help to better understand PV recognition by RNF213. We observed frequent colocalization of RNF213 with type I RH, but not type II *Toxoplasma* PVs, in unprimed cells that express RNF213 at low baseline protein levels. These observations possibly suggest that RH-containing PVs relative to type II PVs are enriched for an unspecified molecular pattern detected by RNF213 with high affinity. *Toxoplasma* strains differ in their repertoire of polymorphic effector proteins (11), and it is intriguing to speculate that differences between polymorphic effector proteins may impact the immunogenicity of PVs. These secreted effector proteins mediate, for example, host mitochondria association with PVs, control nutrient uptake, and impact PV membrane permeability (60–62). Specifically, loss of membrane integrity was previously shown to promote immune recognition of bacteria-containing vacuoles by different intracellular host defense proteins (63, 64). Therefore, secreted polymorphic effectors and their cellular activities are candidate *Toxoplasma*-associated patterns that may be sensed by RNF213. Whether RNF213 directly detects these putative pathogen- or damage-associated molecular patterns present at the *Toxoplasma* PV or instead senses PVs indirectly through auxiliary host factors is yet another question awaiting future examination.

An RNF213 ortholog also exists in mice, and Rnf213-deficient animals are more susceptible to L. monocytogenes and respiratory syncytial virus infections than their wild-type counterparts (55). Whether murine Rnf213 plays a role in *Toxoplasma* PV ubiquitylation, IFN-γ-inducible cell-autonomous immunity, and host protection *in vivo* needs to be tested in the future. Potentially complicating this analysis is the fact that mice are equipped with a defense system executed by IFN-γ-inducible IRGs, which have been shown to be essential for *Toxoplasma* PV ubiquitylation in mouse cells (21, 28, 30). Genes encoding the effector IRGs that control PV ubiquitylation in mouse cells are absent from the human genome (33). Therefore, IRGs are either required for delivering Rnf213 to PVs in mouse but not human cells, or IRGs facilitate the targeting of E3 ligases other than Rnf213 to PVs, thereby executing a host defense pathway that would make Rnf213 obsolete in murine host defense to *Toxoplasma.* To fully address these questions surrounding the role of Rnf213 in murine immunity to *Toxoplasma*, mice deficient for not only Rnf213 but also deficient for both Rnf213 and the IRG system would need to be examined.

Regardless of the role Rnf213 plays in mice, our study establishes RNF213 as a potent executioner of cell-autonomous immunity to *Toxoplasma* in human cells. We show that in A549 and HFF-1 cells, RNF213 is robustly induced by IFN-γ, a cytokine essential for murine host defense to *Toxoplasma in vivo* (65–67). Whereas the importance of IFN-γ in murine immunity to *Toxoplasma* infection is clearly established, it has been shown that humans with IFN-γ receptor deficiencies display normal resistance to *Toxoplasma* infections (68). Several lines of evidence suggest that proinflammatory signaling molecules, including TNF-α and CD40L, can compensate for the absence of IFN-γ signaling and are sufficient on their own to induce robust cell-autonomous immunity in human blood monocytes, macrophages, and microglia cells (68–71). It is worth mentioning that TNF-α was shown to activate RNF213 transcription in human endothelial cells (72), raising the distinct possibility that RNF213 expression is a central defense node activated by multiple inducers of cell-autonomous immunity to *Toxoplasma* in human cells. Therefore, investigating the contribution of RNF213 to antiparasitic host defense in diverse human cell and tissue types, as well as the role of upstream immune signals other than IFN-γ, are important areas of future research.

## MATERIALS AND METHODS

### Cell lines and culture.

Cell lines used for this study include A549 (ATCC CCL-185), Vero cells (ATCC CCL-81), and HFF-1 cells (ATCC SCRC-1041). A549 and Vero cells were cultured in Dulbecco’s modified Eagle medium (DMEM) containing 10% heat-inactivated FBS, MEM nonessential amino acids (NEAA; Gibco), and β-mercaptoethanol (BME; Gibco). HFF-1 cells were cultured in DMEM containing 15% heat-inactivated FBS, NEAA, BME, and GlutaMax (Gibco). ISG15 knockout (KO) cells and their corresponding parental A549 cell line were kindly provided by David Sibley (Washington University in St. Louis) (50). hGBP1 KO cells and their corresponding parental A549 cell line were kindly provided by Eva Frickel (University of Birmingham) (48). All cells were grown at 37°C in 5% CO_2_.

### Cell line editing.

KO A549 cell lines for HOIP, HOIL-1, and RNF213 cells were generated by the Duke Functional Genomics core using CRISPR/Cas9 technology. Single guide RNAs (sgRNAs) were designed using CHOPCHOP (73) and Cas-OFFinder (74) cloned into PX459 V2 (Addgene; plasmid no. 62988) (75). sgRNA-PX459 constructs were transfected into A549, and after 24 h, cells were selected with 2 μg/mL puromycin (Sigma) for 3 days. For HOIP, the sgRNAs were designed to target 5′-GCAGCGCCAAGACAAGATGC-3′ (clone 1) and 5′- GTTCCATATGTGAGCGACCT-3′ (clone 2). For HOIL-1, the sgRNA was designed to target 5′-AGTGCGCCCTGATATGACAG-3′ (clones 1 and 2). For RNF213, sgRNAs were designed to target 5′-GTGGACCGATTTGCAGTACA-3′ (pool 1), 5′-CACGTGGTACCATTGCCGGA-3′ (pool 2), and 5′-CGTCTTCATCGGCTACCACT-3′ (pool 3). HOIP and HOIL-1 KO clones were obtained from pools by single-cell serial dilutions and genotyped by Western blotting. For RNF213, pooled cells exhibited highly efficient genome editing (>95%) as measured by PCR and were used for all subsequent experiments.

### Toxoplasma gondii strains and passaging.

The Toxoplasma gondii strains used for this study include green fluorescent protein (GFP)-expressing type I (RH) and type II (Pru A7), and GFP-click-beetle luciferase expressing type I (RH-Luc) and type II (Me49-Luc). GFP-expressing stains were a gift from John Boothroyd (Stanford University) (62, 76), and luminescent strains were a gift from Jeroen Saeiji (University of California) (77). All strains were passaged in monolayers of Vero cells as previously described (28, 36, 48). T. gondii tachyzoites were harvested by scraping cell monolayers and lysing with a 27.5G syringe. To get rid of cell host debris and aggregated parasites, lysates were centrifuged at least three times for 1 min at 1,000 rpm and passed through a 5-μm-pore-size filter. The lysate was then centrifuged for 10 min at 1,800 rpm, and tachyzoites were resuspended in DMEM supplemented with 10% FBS, NEAA, and BME for further experimentation.

### *Toxoplasma* infections.

All infections were carried out in cells either primed with IFN-γ (100 U/mL) for 18 to 24 h or left unprimed. All infections were carried out as previously described (28, 36, 48). After the addition of tachyzoites, cells were centrifuged at 1,000 rpm at room temperature for 5 min and grown at 37°C in a CO_2_ incubator for 2 h. Cells were then washed two times with Hanks’ balanced salt solution (HBSS; Gibco), and fresh medium was added and left to incubate for the remainder of the experiment. For immunofluorescent microscopy, cells were plated on glass coverslips, infected at a multiplicity of infection (MOI) of 3, and fixed at indicated time points. For parasites per vacuole, cells were infected at an MOI of 2 for type II (A7 Pru) and an MOI of 1 for type I (RH) and fixed at 24 hpi for imaging. For luminescent burden assays, cells were infected at an MOI of 2 with type II (Me49-Luc) and at an MOI of 1 with type I (RH-Luc), and luciferase activity was measured at 24 hpi.

### Plaque assays.

For plaque assays, a confluent layer of A549 cells were plated in 96-well plates, and cells were either left unprimed or primed with IFN-γ (100 U/mL) for 18 to 24 h. For infection, 50 tachyzoites were added to each well and centrifuged at 1,000 rpm at room temperature for 5 min to synchronize the infection. At 2 hpi, cells were washed two times with HBSS, and fresh medium was added. At 72 hpi, plaques were live-imaged and quantified for each well.

### Luciferase assays.

For assessing NF-κB activity, cells were cotransfected with 4× NF-κB-Luc, provided by Johannes A. Schmid (Addgene; plasmid no. 111216), and pGL4.74-hRluc/TK (Promega) using the transfection reagent FuGene HD (Promega). Cells were treated with TNF-α (100 ng/mL) for 24 h and lysed using passive lysis 5× buffer (Promega). Cell extracts were prepared for measuring luciferase activity using the dual-luciferase reporter assay system according to the manufacturer’s instructions (Promega). For assessing burden with luciferase-expressing strains, at 24 hpi, cells were lysed with passive lysis 5× buffer (Promega) followed by addition of luciferin for measuring luciferase activity. All measurements were done with the EnSpire 2300 multilabel reader (PerkinElmer).

### Western blotting.

For all Western blotting, protein samples were harvested on ice with 200 μL of radioimmunoprecipitation assay (RIPA) buffer (Sigma) containing 1× of protease inhibitor cocktail (Sigma; catalog no. P8340) and 4 U/mL of DNase I (NEB) and incubated at 4°C for 30 min. Samples were centrifuged at 14,500 rpm and 4°C for 10 min, mixed with 2× Laemmli buffer (Bio-Rad) containing 5% β-mercaptoethanol, and incubated at 95°C for 10 min. For RNF213 immunoblotting, samples were incubated at 56°C for 10 min. Samples were run on 4 to 20% mini-Protean TGX stain-free gels (Bio-Rad) and transferred to polyvinylidene difluoride (PVDF) membranes (Bio-Rad) using the Trans-Blot Turbo transfer system (Bio-Rad). For RNF213 immunoblotting, samples were transferred overnight at 4°C using a tank transfer system. Membranes were blocked with 5% nonfat dry milk (Bio-Rad) in Tris-buffered saline (TBS) containing 0.1% Tween 20 and probed with the following: rabbit monoclonal anti-GBP1 (1:5,000; Abcam; catalog no. ab131255), rabbit polyclonal anti-ISG15 (1:1,000; ProteinTech; catalog no. 15981-1-AP), rabbit polyclonal anti-RNF213 (1:2,000; Sigma; Human Protein Atlas no. HPA026790), rabbit polyclonal anti-HOIP (1:1,000; Abcam; catalog no. ab46322), mouse monoclonal anti-HOIL-1, clone 2E2 (1:1,000; Sigma; catalog no. MABC576), and rabbit polyclonal anti-GAPDH (glyceraldehyde-3-phosphate dehydrogenase) (1:5,000; Abcam; catalog no. ab9485).

### Immunofluorescent microscopy.

All cells used for microscopy were seeded on glass coverslips. Infected cells were fixed with 4% paraformaldehyde followed by three phosphate-buffered saline (PBS) washes. For ubiquitin and TAX1BP1 immunostaining, cells were permeabilized with ice-cold methanol for 1 min followed by three PBS washes. For any other immunostaining, cells were permeabilized with 0.05% saponin in blocking buffer (5% bovine serum albumin [BSA], 2.2% glycine, and 0.05% saponin in PBS). All cells were blocked for 30 min at room temperature and probed overnight at 4°C with the following: rabbit polyclonal anti-RNF213 (1:1,000; Sigma; Human Protein Atlas no. HPA003347), mouse monoclonal anti-ubiquitin, clone FK2 (1:200; Cayman Chemical, catalog no. 14220, and Sigma, catalog no. 04-263), rabbit monoclonal anti-linear ubiquitin, clone 1E3 (1:200; Sigma; catalog nos. MABS199 and ZRB2114), rabbit monoclonal anti-K63 ubiquitin, clone Apu3 (1:100; Sigma; catalog no. 05-1308), rabbit monoclonal anti-K48 ubiquitin, clone Apu2 (1:500; Sigma; catalog no. ZRB2150), rabbit polyclonal anti-p62 (1:500; MBL; catalog no. PM045), rabbit polyclonal anti-NDP52 (1:200; Abnova; catalog no. H00010241-D01), rabbit polyclonal anti-optineurin (1:200; ProteinTech; catalog no. 10837-1-AP), and rabbit polyclonal anti-TAX1BP1 (1:100; Abcam; catalog no. ab121812). The next day, cells were washed three times with 0.05% saponin in PBS and probed with Alexa Fluor-conjugated secondary antibodies (1:1,000; Invitrogen) and nuclear dye Hoechst 33258 (2 μg/mL; Invitrogen). Coverslips were mounted on glass slides with 1:9 mixture of *p*-phenylenediamine (PPD) and Mowiol 4-88 and allowed to cure overnight. Images for quantification were acquired on a Zeiss Axio Observer.Z1 epifluorescence microscope using AxioVision 4.8. For each sample, six to eight randomly chosen fields of view at ×63 magnification and more than 100 Toxoplasma gondii PVs were imaged. Representative images were acquired on a Zeiss 880 Airyscan Fast inverted confocal microscope using ZEN software (Zeiss). Samples were blinded during digital quantitation using the Blind Analysis tool (https://imagej.net/plugins/blind-analysis-tools). Targeting was defined as having >50% intense protein signal surrounding the PV.

### Statistical analysis.

Graphs and statistics were generated in GraphPad Prism 9. All data represents mean ± the standard error of the mean (SEM) of three to four independent experiments. Statistical significance was calculated using one-way or two-way analysis of variance (ANOVA) followed by Tukey’s multiple-comparison test as indicated. Significance was defined as follows: ∗, *P < *0.05; ***, *P < *0.01; ***, *P < *0.001; ****, *P < *0.0001; and ns, not significant.
