# Supplementary material for: Interferon-Inducible E3 Ligase RNF213 Facilitates Host-Protective Linear and K63-Linked Ubiquitylation of Toxoplasma gondii Parasitophorous Vacuoles
Source: mBio. 2022 Sep 26;13(5):e01888-22. doi: 10.1128/mbio.01888-22 (PMC9601232; doi:10.1128/mbio.01888-22)
Supplement: FIG S1 [file mbio.01888-22-s0001.pdf]

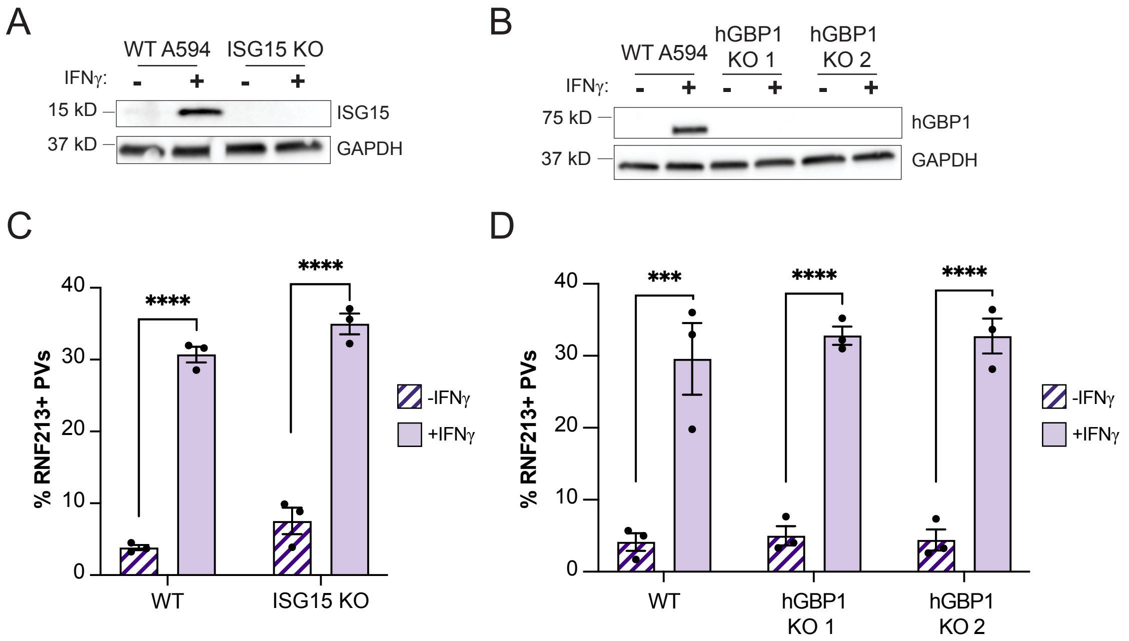

Fig. S1. RNF213 is recruited to Toxoplasma PVs independent of ISG15 and GBP1. (A-B) Western blotting for ISG15 and GBP1 protein expression in unprimed and IFNγ-primed (100 U/mL) WT, ISG15 KO, and GBP1 KO A549 cells. **(C-D)** Unprimed and IFNγ-primed (100 U/ mL) WT, ISG15 KO, and GBP1 KO A549 cells were infected with Toxoplasma type II Pru at an MOI of 3 and RNF213 recruitment to Toxoplasma PVs was quantified at 6 hpi. All data depict the mean ± SEM from 3 independent experiments. 2-way ANOVA followed by Tukey's multiple comparison test was used to determine significance. *= $p < 0.05$, **= $p < 0.01$, ***= $p < 0.001$, ****=$p < 0.0001$; n.s.=not significant.
