# Supplementary material for: Interferon-Inducible E3 Ligase RNF213 Facilitates Host-Protective Linear and K63-Linked Ubiquitylation of Toxoplasma gondii Parasitophorous Vacuoles
Source: mBio. 2022 Sep 26;13(5):e01888-22. doi: 10.1128/mbio.01888-22 (PMC9601232; doi:10.1128/mbio.01888-22)
Supplement: FIG S2 [file mbio.01888-22-s0002.pdf]

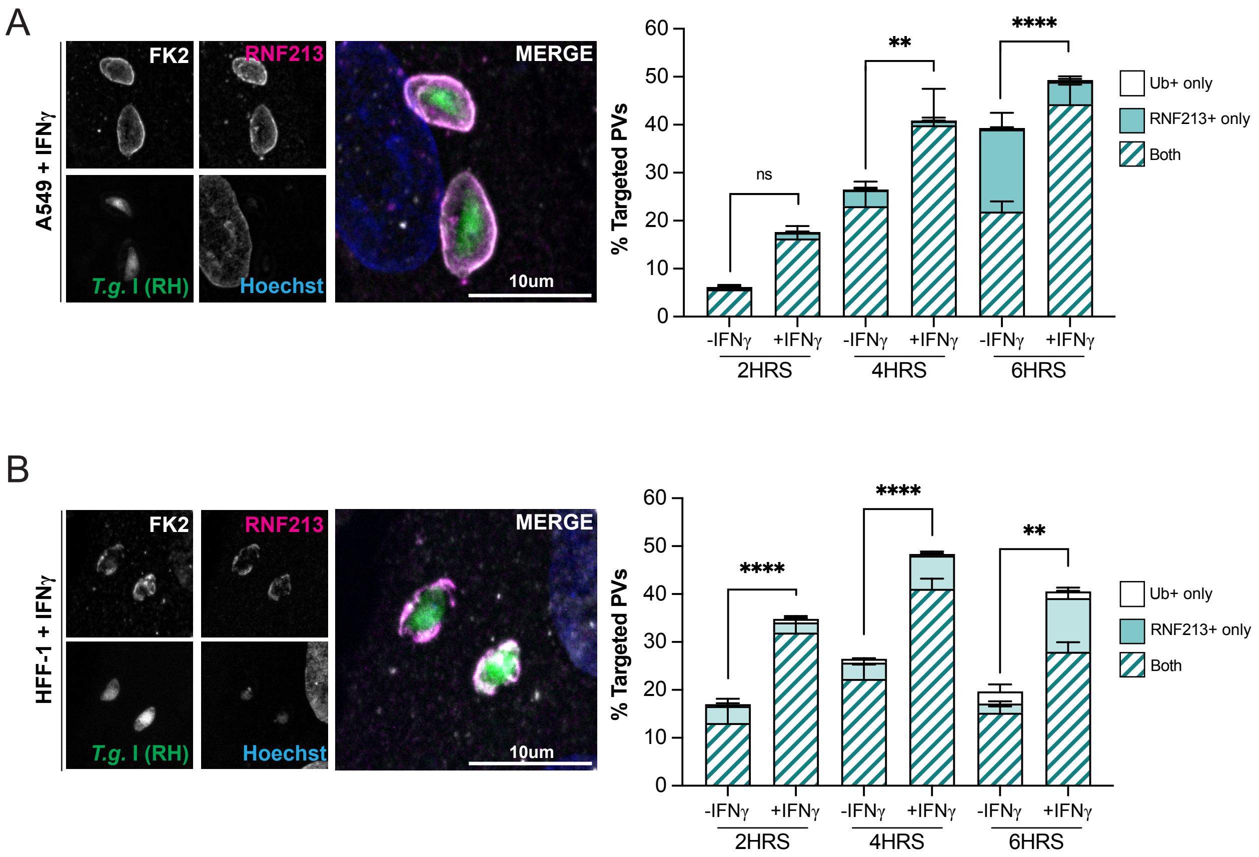

Fig. S2. RNF213 co-localizes with ubiquitin on Toxoplasma RH PVs. Unprimed and IFNγ-primed (100 U/mL) WT A549 **(A)** and HFF-1 **(B)** cells were infected with Toxoplasma Type I (RH) at an MOI of 3 and RNF213 recruitment to PVs and co-localization with ubiquitin (FK2) was quantified at 2 hpi, 4 hpi, and 6 hpi. Statistical comparisons is shown for groups "Both". Representative confocal images at 6 hpi are shown. All data depict the mean ± SEM from 3-4 independent experiments. 2-way ANOVA followed by Tukey's multiple comparison test was used to determine significance.* = p <0.05, **= p <0.01, ***= p <0.001, ****=p<0.0001; n.s.=not significant.
