# Supplementary material for: Interferon-Inducible E3 Ligase RNF213 Facilitates Host-Protective Linear and K63-Linked Ubiquitylation of Toxoplasma gondii Parasitophorous Vacuoles
Source: mBio. 2022 Sep 26;13(5):e01888-22. doi: 10.1128/mbio.01888-22 (PMC9601232; doi:10.1128/mbio.01888-22)
Supplement: FIG S3 [file mbio.01888-22-s0003.pdf]

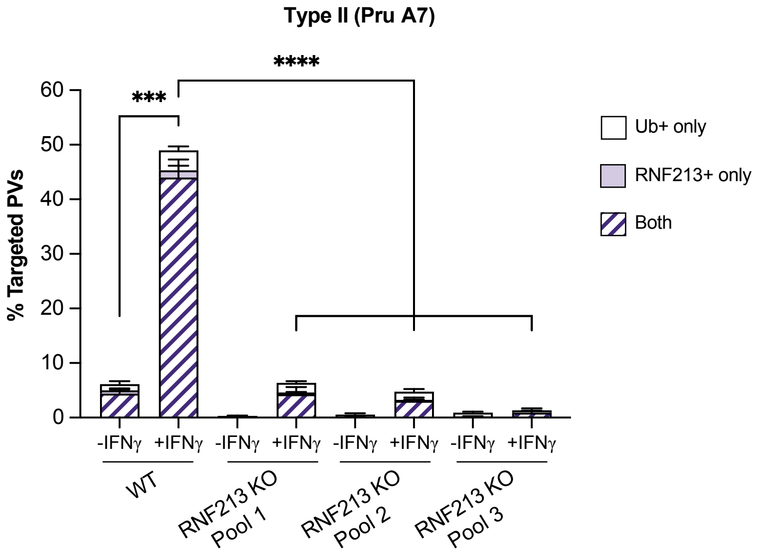

Fig. S3. Residual RNF213 co-localizes with ubiquitin in RN213 KO pools. Unstimulated or IFNγ-primed (100 U/mL) WT cells and RNF213 KO pools cells were infected with T.g. Type II (Pru) at an MOI of 3. RNF213 recruitment to the PV and ubiquitin (FK2) co-localization was quantified at 6 hpi. Statistical comparisons is shown for groups "Both". All data depict the mean ± SEM from 3 independent experiments. 2-way ANOVA followed by Tukey's multiple comparison test was used to determine significance. 2-way ANOVA followed by Tukey's multiple comparison test was used to determine significance.*= p <0.05, **= p <0.01, ***= p <0.001, ****=p<0.0001; n.s.=not signifi-
