# Supplementary material for: Interferon-Inducible E3 Ligase RNF213 Facilitates Host-Protective Linear and K63-Linked Ubiquitylation of Toxoplasma gondii Parasitophorous Vacuoles
Source: mBio. 2022 Sep 26;13(5):e01888-22. doi: 10.1128/mbio.01888-22 (PMC9601232; doi:10.1128/mbio.01888-22)
Supplement: FIG S4 [file mbio.01888-22-s0004.pdf]

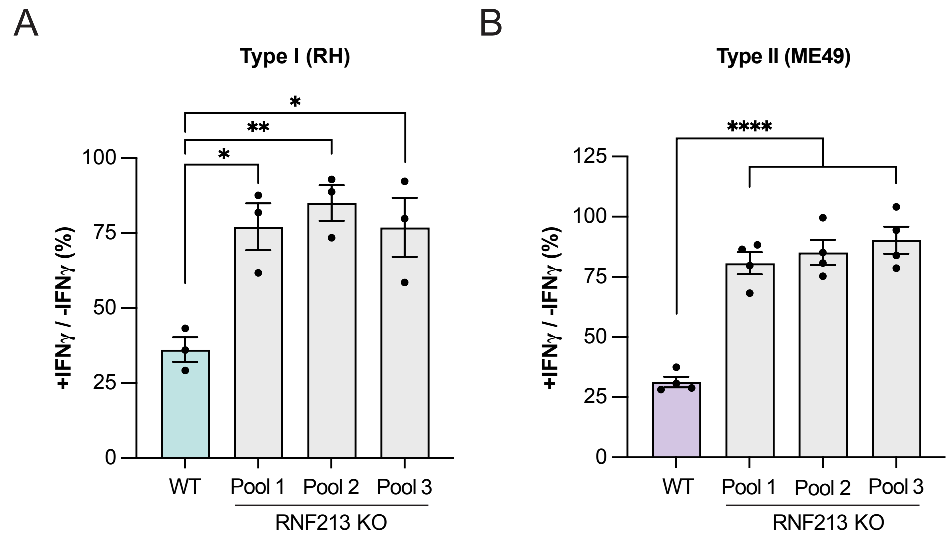

Fig. S4. RNF213 mediates restriction of Toxoplasma type I RH and type II Me49. Unprimed and IFN γ-primed (100 U/mL) WT and RNF213 KO A549 cells were infected with luciferase expressing strains RH **(A)** and Me49 **(B)** at an MOI of 1 or MOI of 2, respectively, and cell lysates were analyzed for luciferase activity at 24 hpi. Growth of each strain in IFNγ-primed cells is normalized to growth in unprimed cells. All data depict the mean ± SEM from 3-4 independent experiments. One-way ANOVA followed by Tukey's multiple comparison test was used to determine significance. *= $p < 0.05$, **= $p < 0.01$, ***= $p < 0.001$, ****=$p < 0.0001$; n.s.=not significant.
