# Supplementary material for: Interferon-Inducible E3 Ligase RNF213 Facilitates Host-Protective Linear and K63-Linked Ubiquitylation of Toxoplasma gondii Parasitophorous Vacuoles
Source: mBio. 2022 Sep 26;13(5):e01888-22. doi: 10.1128/mbio.01888-22 (PMC9601232; doi:10.1128/mbio.01888-22)
Supplement: FIG S5 [file mbio.01888-22-s0005.pdf]

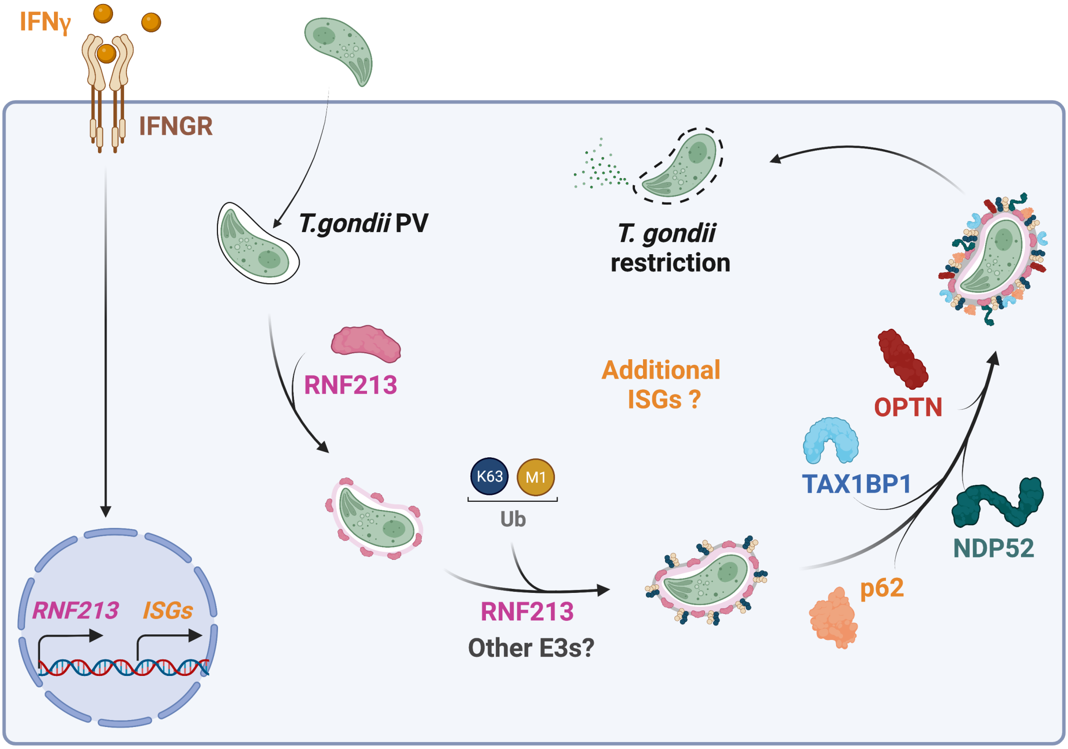


**Fig. S5. Schematic for RNF213-mediated restriction of *Toxoplasma* growth.** RNF213 localizes to *Toxoplasma* PVs and facilitates K63-linked and linear PV ubiquitylation. This process is independent of LUBAC but may still require additional ubiquitin E3 ligases.. Ubiquitin adaptor proteins p62, TAX1BP1, NDP52, and OPTN associate with ubiquitylated PVs. RNF213-mediated PV ubiquitylation is unlikely to be sufficient for restriction of *Toxoplasma* growth but rather appears to require additional ISGs. Created with BioRender.com
